# Hypercapnic respiratory distress and loss of consciousness: a complication of supraclavicular brachial plexus block

**DOI:** 10.1186/s40981-015-0014-5

**Published:** 2015-09-09

**Authors:** Yuka Sakuta, Naoko Kuroda, Masatsugu Tsuge, Yoshihisa Fujita

**Affiliations:** 1 Department of Anesthesiology & ICM, Kawasaki Medical School Hospital, 577 Matsushima, Kurashiki, Okayama 7010192 Japan; 2 Student in the postgraduate course, Kawasaki Medical School, 577 Matsushima, Kurashiki, Okayama 7010192 Japan

**Keywords:** Supraclavicular brachial plexus block, Ultrasound, Hemidiaphragmantic palsy

## Abstract

Supraclavicular brachial plexus block is a common anesthetic technique performed for surgery of the upper extremities. We experienced a case of acute hypercapnic respiratory distress with loss of consciousness during creation of an arteriovenous fistula under ultrasound-guided supraclavicular brachial plexus block using 30 mL of 0.75 % ropivacaine. We detected ipsilateral hemidiaphragmatic paralysis by means of M-mode ultrasonography of the block. We thus speculate that phrenic nerve palsy caused by supraclavicular brachial plexus block was the underlying mechanism of the event. Bedside ultrasonography played a pivotal role in making a differential diagnosis and in managing this patient.

## Background

Phrenic nerve paralysis is known to anesthesiologists as a common complication associated with interscalene brachial plexus block [1], while it is often ignored after supraclavicular brachial plexus block [2]. We present the case of a patient with ipsilateral hemidiaphragmatic paralysis after supraclavicular brachial plexus block, which manifested as loss of consciousness and hypercapnic respiratory distress. Bedside ultrasonography played an important role in diagnosing hemidiaphragmatic paralysis and managing this patient.

## Case presentation

A 78-year-old-man with chronic renal failure had been on hemodialysis via an arteriovenous fistula on his left arm three times a week for 4 years. He was scheduled for revision of an arteriovenous fistula on the right arm because of occlusion of the fistula. He had a prosthetic mitral valve and an intravenous pacemaker that had been set to 65 beats per minute in ventricular back-up mode for 15 years. The pacemaker had been implanted because he had suffered from acute mitral regurgitation due to rupture of the chordae tendinae associated with myoxomatous degeneration of the mitral leaflets and sick sinus syndrome 2 months after the prosthetic mitral valve surgery. The patient’s medical history also included hypothyroidism and chronic hepatitis B with thrombocytopenia and splenomegaly but not chronic obstructive lung diseases (COPD). Preoperative transthoracic echocardiography (TTE) revealed normal left ventricular (LV) ejection fraction (EF = 53 %) with a restrictive pattern of LV inflow, as indicated by a 2.3 ratio of early transmitral inflow peak velocity to atrial transmitral inflow peak velocity, suggesting elevated left atrial pressure. Moderate pulmonary hypertension was diagnosed, with a 48-mmHg tricuspid valve pressure gradient calculated from the tricuspid regurgitant jet velocity. There were no abnormalities in the native aortic valve or the prosthetic mitral valve.

**Fig. 1:**
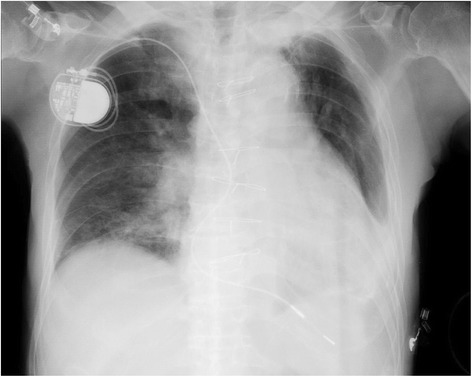
Portable supine chest radiograph in the operating room. It shows diffuse hilar haziness with increased density at the base of the right and left lungs. There were no signs for pneumothorax and a slight elevation of the right hemidiaphragm. Although the right hemidiaphragm was located at the fourth intercostal space, the right hemidiaphragmatic paralysis could not be diagnosed on this chest radiograph

In the operating room, we started continuous electrocardiogram (ECG) on standard lead II, automated noninvasive measurement of blood pressure every 5 min, and measurement of oxygen saturation by pulse oximetry (SpO_2_). A right supraclavicular brachial plexus block was performed under ultrasound guidance with 30 mL of 0.75 % ropivacaine. After confirming the anesthetic effect of the block, surgery was started 30 min later under the observation of by a registered nurse who worked in the operating rooms. While no sedatives were administered during surgery, the patient was given supplementary oxygen at 3 l per minute via a face mask during the surgery because he reported some difficulty breathing 20 min after injection of the local anesthetic. His complaint resolved soon thereafter. The patient’s blood pressure was stable between 160/75 mmHg and 170/80 mmHg, his heart rate was 70–80 beats per minute, and SpO_2_ was between 96–100 %. Although an arteriovenous fistula was created within approximately 80 min as planned, inadequate blood flow of the fistula was detected by Doppler ultrasound. The patient consented to immediately undergoing the surgery a second time. About 10 min after surgery began again, he complained of dyspnea and nausea, accompanied by a decrease in SpO_2_ to 70 %. Oxygen flow was increased to 10 l per minute. Five minutes after that, his SpO_2_ decreased further to 50 %, and he was unresponsive to verbal commands.Anesthesiologists were called at this time. The patient was unresponsive to verbal and painful stimuli, and his arterial pulse could be palpated easily. Because his breath sounds were diminished, *we assisted his ventilation via a bag-mask device, followed by placement of a #5 laryngeal mask airway (LMA)*. Ventilatory support was continued with 100 % oxygen. SpO_2_ returned to 100 % soon thereafter, but the patient remained unconscious with stable blood pressure of 155/80 mmHg and heart rate of 75 beats per minute. Gas analysis of a blood sample from the femoral artery showed pH 6.93, pCO_2_ 121 mmHg, and pO_2_ 98 mmHg. We speculated that the loss of consciousness was caused by acute hypercapnia, while the underlying cause remained undetermined. We decided to abort interrupt the surgery, and the surgical wound was closed. A portable supine chest radiograph was not remarkable as compared with the preoperative one, showing hilar haziness of the lung field, no signs for pneumothorax, and a slight elevation of the right hemidiaphragm, with increased density at both lung bases. These findings were compatible with pulmonary congestion (Fig. 1).

**Fig. 2:**
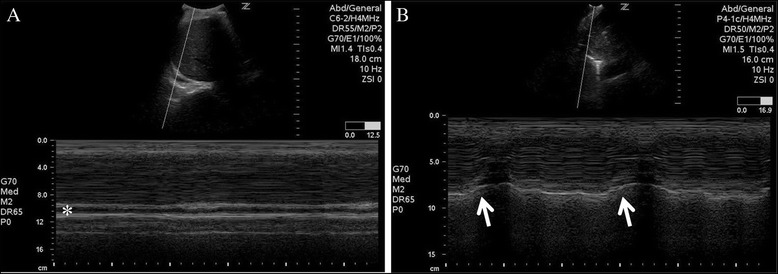
M-mode ultrasound images during respiratory cycles. The right image (**a**) demonstrated loss of respiratory movement of the right hemidiaphragm, while in the left image (**b**), normal respiratory movement of the left hemidiaphragm was detected (arrows inspiratory phase). A small pleural effusion (aechoic space) was seen posterior to the diaphragm (asterisk)

We performed a bedside ultrasound examination to identify the cause of the acute hypercapnic respiratory distress. No apparent changes compared to the preoperative recording were detectable by TTE, including visually estimated LVEF (approximately 55 %). There was lung sliding with A-lines and a normal B-line pattern on the right and left anterior chest walls, confirming the absence of pneumothorax on the chest roentgenogram and ruling out the pulmonary congestion suggested by the chest roentgenogram, respectively. We found that there was no movement of the right diaphragm during the respiratory cycle, while normal respiratory movement of the left diaphragm was visualized by M-mode ultrasound imaging (Fig. 2). A diagnosis of right hemidiaphragmatic paralysis caused by phrenic nerve palsy was made. We canceled further examinations such as computed tomography and magnetic resonance imaging. The patient was transferred to the intensive care unit, and positive pressure ventilation was continued for respiratory support via the LMA at 40 % F_i_O_2_. Spontaneous breathing became increasingly stronger, and he gained full consciousness after 2 h. The LMA was removed, and ventilator support was terminated.

**Fig. 3:**
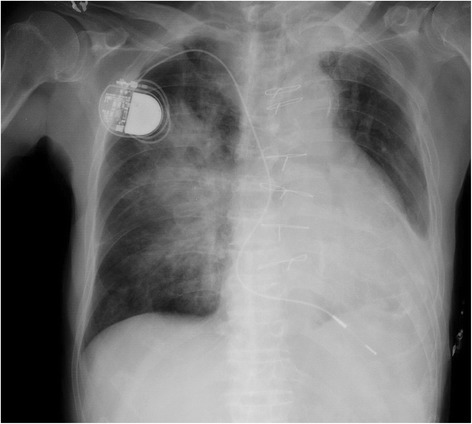
Portable chest radiograph the next day in the ICU. The patient was in the supine position. Diffuse hilar haziness remained. The right hemidiaphragm had moved to the sixth intercostal space, suggesting restoration of right hemidiaphragmatic function

We confirmed restoration of respiratory movement of the right hemidiaphragm by means of ultrasonography. The next day he was uneventfully transferred to the ward. A portable chest roentgenogram on that day showed that the right hemidiaphragm was shifted downward, suggesting the restoration of normal diaphragmatic function (Fig. 3). A new arteriovenous fistula was uneventfully created under supraclavicular brachial plexus block with 20 mL of 0.75 % ropivacaine 1 week later.

### Discussion

We presented a case of hypercapnic respiratory distress and loss of consciousness resulting from transient phrenic nerve palsy associated with supraclavicular brachial plexus block. Interscalene brachial plexus block almost invariably causes ipsilateral phrenic nerve palsy [1]. However, it is thought to occur less frequently with brachial plexus block via the supraclavicular approach, depending on the amount of local anesthetic used and the techniques employed (e.g., nerve stimulator or ultrasound guidance). Respiratory symptoms are uncommon during the brachial plexus block irrespective of the approaches [3].

We detected hemidiaphragmatic paralysis in this patient with hypercapnic respiratory distress by means of bedside ultrasonography. There have been few ultrasonographically documented cases of hemidiaphragmatic palsy after supraclavicular brachial plexus block.

Hemidiaphragmatic paralysis is usually asymptomatic at rest, because it is compensated for by other respiratory muscles; at most, it may lead to limitations in exercise [4]. Urmey et al. [1] investigated the effects of hemidiaphragmatic paralysis on pulmonary function during interscalene brachial plexus block and concluded that the block should not be performed in patients who are dependent on intact diaphragmatic function. Hemidiaphragmatic paralysis causes decreased values on pulmonary function tests, such as forced vital capacity, forced expiratory volume, and peak expiratory flow rate [5]. In addition, it causes abdominal expansion during inspiration, leading to regional hypoventilation in the ipsilateral lower lung and a reduction in gas exchange. These changes are usually compensated for by recruitment of the intercostal or accessory muscles [6]. However, they may become symptomatic, causing dyspnea or hypoventilation in the supine position because of enhanced shift of the paralyzed diaphragm toward the head [4]. In addition to the supine position, there are at least two other possible explanation for the development of hypercapnic respiratory distress with loss of consciousness in this patient. First, limited cardiopulmonary reserve may be responsible, as evidenced by the patient’s implanted prosthetic mitral valve and pacemaker and his advanced age of 86 years [4]. Second, chronic renal failure itself may be a high risk. Afonso et al. [7] reported three consecutive cases of respiratory arrest necessitating tracheal intubation in patients undergoing arteriovenous graft placement with supraclavicular brachial plexus block; however, the exact underlying causes other than chronic renal failure and obesity could not be delineated. The authors suggested that chronic renal failure may represent a high-risk group for respiratory failure after supraclavicular brachial plexus block.

Oxygen administered via a face mask may have transiently relieved the patient’s respiratory symptoms when he had difficulty breathing soon after brachial plexus block. We speculate that this may have resulted in further depression of respiration and thus in hypercapnia and, consequently, loss of consciousness. Recognition of risk factors for developing symptomatic hemidiaphragmatic paralysis in this patient or earlier examination with ultrasound might have led us to avoid brachial plexus block or to initiate ventilatory support earlier instead of administering oxygen via face mask.

Bedside ultrasound plays a pivotal role in the management of acute respiratory distress and in finding its underlying causes [8]. M-mode recording of the diaphragmatic dome through a respiratory cycle is an easy way to document a nonfunctioning diaphragm, while in the present case, the supine chest roentgenogram was not conclusive for the diagnosis of hemidiaphragmatic paralysis. Absence of thickening of the diaphragm at the zone of apposition with the rib base is an alternative ultrasonographic technique for the diagnosis of diaphragmatic paralysis [4].

Point-of-care ultrasound is becoming an essential diagnostic skill for all physicians [9]. Anesthesiologists routinely use ultrasound machines for procedures such as catheter placement or nerve blocks, as well as transesophageal echocardiography for cardiac surgery. Thus, they are familiar with ultrasound machines and should therefore be able to improve patient care by the addition of diagnostic ultrasonography. Training to ensure competent use of this technology is thus of utmost importance [8, 9].

## Conclusions

A decreased cardiopulmonary reserve, advanced age, and, possibly, chronic renal failure may have been risk factors in this patient for developing hypercapnic respiratory distress and loss of consciousness with supraclavicular brachial plexus block. Bedside ultrasonography was very useful in establishing the correct diagnosis; it allowed for ventilator support until the return of diaphragmatic function and precluded unnecessary examinations such as brain computed tomography or magnetic resonance imaging.

## Consent

Patient’s family reviewed the case report and gave written permission for the authors to publish it.

## Abbreviations

COPD: chronic obstructive lung diseases
ECG: electrocardiogram
EF: ejection fraction
LMA: laryngeal mask airway
LV: left ventricular
SpO_2_: oxygen saturation by pulse oximetry
TTE: transthoracic echocardiography
